# Peripartale Blutungen, Diagnostik und Therapie: Update der S2k-Leitlinie AWMF 015/063 vom August 2022

**DOI:** 10.1007/s00101-022-01224-6

**Published:** 2022-11-25

**Authors:** T. Annecke, H. Lier, T. Girard, W. Korte, G. Pfanner, D. Schlembach, O. Tiebel, C. von Heymann

**Affiliations:** 1 Klinik für Anästhesiologie und operative Intensivmedizin, Klinikum Köln-Merheim, Kliniken Köln, Universität Witten/Herdecke, Ostmerheimer Str. 200, 51109 Köln, Deutschland; 2 Medizinische Fakultät und Uniklinik Köln, Klinik für Anästhesiologie und Operative Intensivmedizin, Universität zu Köln, Köln, Deutschland; 3 Klinik für Anästhesiologie, Universitätsspital Basel, Basel, Schweiz; 4 Hämostase- und Hämophiliezentrum, Zentrum für Labormedizin Sankt Gallen, Sankt Gallen, Schweiz; 5 Anästhesie und Intensivmedizin, Landeskrankenhaus Feldkirch, Feldkirch, Österreich; 6 Klinik für Geburtsmedizin, Vivantes Klinikum Neukölln, Berlin, Deutschland; 7 Institut für Klinische Chemie und Laboratoriumsmedizin, Universitätsklinikum Dresden, Dresden, Deutschland; 8 Klinik für Anästhesie, Intensivmedizin, Notfallmedizin und Schmerztherapie, Vivantes Klinikum im Friedrichshain, Berlin, Deutschland

**Keywords:** Geburtshilfliche Hämorrhagie, Leitlinie, Gerinnungstherapie, Schock, Intensivmedizin, Obstetric hemorrhage, Guidelines, Coagulation treatment, Shock, Intensive care medicine

## Abstract

Anhand einer fiktiven Kasuistik wird die aktuelle Leitlinie „Peripartale Blutungen, Diagnostik und Therapie“ mit einem Schwerpunkt auf die anästhesiologische Sicht zusammengefasst. Die aktualisierte Leitlinie wurde unter Federführung der Deutschen Gesellschaft für Gynäkologie und Geburtshilfe und Beteiligung weiterer Fachgesellschaften und Interessenvertretungen aus Deutschland, Österreich und der Schweiz erarbeitet und bei der AWMF unter der Registriernummer 015/063 publiziert.

## Abstract (en)

The current S2k guidelines on the diagnostics and treatment of peripartum hemorrhage are summarized in this article from the perspective of anesthesiology based on a fictitious case report. The update of the guidelines was written under the auspices of the German Society of Gynecology and Obstetrics with the participation of other professional societies and interest groups from Germany, Austria and Switzerland and published by the AWMF in 2022 under the register number 015/063.

## Präambel

Peripartale Hämorrhagien (PPH) gehören zu den häufigsten Notfallsituationen im Kreißsaal und tragen signifikant zur weltweiten Müttersterblichkeit bei. Prospektive Untersuchungen mit objektiven Messungen zeigen verstärkte Blutungen bei bis zu 10 % aller Entbindungen [1]. Etwa 3 % aller Entbindungen sind von einem Blutverlust ≥ 1500 ml betroffen. Um die aktuelle Evidenz abzubilden, wurde die AWMF-Leitlinie 015/063 „Peripartale Blutungen, Diagnostik und Therapie“ auf S2K-Niveau von einer Expertengruppe unter Beteiligung der relevanten Fachgesellschaften und Interessenvertreter aus Deutschland, Österreich und der Schweiz mit formaler Konsensfindung aktualisiert und im August 2022 verabschiedet.

In der folgenden Übersichtsarbeit sollen anhand eines fiktiven Fallbeispiels wichtige anästhesiologische Aspekte der neuen Leitlinie behandelt werden. Für eine ausführliche Darstellung aller behandelten Themenbereiche sei der geneigte Leser auf die AWMF-Originalpublikation verwiesen [2].

## Kasuistik: Geburtsverlauf

*Eine 35-jährige adipöse Schwangere (Body Mass Index [BMI]* *37 kg/m*^*2*^*) ohne weitere Vorerkrankungen wird in der 40. SSW nach unauffälligem Schwangerschaftsverlauf zur Entbindung in die geburtshilfliche Abteilung eines Krankenhauses der Grund- und Regelversorgung (Geburtshilfliches Zentrum Level III) aufgenommen. Die Patientin ist bereits Mutter von 4 Kindern. Zur geburtshilflichen Analgesie wurde leitlinienkonform ein lumbaler Periduralkatheter gelegt. Nach unauffälligem Geburtsverlauf und Entwicklung eines lebensfrischen Neugeborenen werden über den liegenden periphervenösen Zugang 3* *IE Oxytocin als Kurzinfusion verabreicht*.

## Leitlinie: Prävention einer PPH

Im ersten Teil der überarbeiteten Leitlinie werden ausführlich Strategien zur Risikostratifizierung und Prävention unter besonderer Berücksichtigung von Plazentalösungs- und Plazentationsstörungen dargestellt. Viele Frauen mit PPH weisen allerdings im Vorfeld keinerlei Risikofaktoren auf. Vorangegangene Kaiserschnitte, Abrasiones, IVF-Maßnahmen, Nikotinabusus und auch Mehrlingsschwangerschaften erhöhen das Risiko für eine Placenta praevia in den Folgeschwangerschaften. Generell nimmt die Inzidenz dieser Störungen zu. Neben der Anamnese besitzt die Ultraschalluntersuchung im II. Trimenon zur Identifikation einer Placenta praevia und von Plazentaimplantationsstörungen („placenta accreta spectrum disorder“, PAS) besondere Relevanz. Werden Risikofaktoren für eine PPH identifiziert, sollte den Empfehlungen der Leitlinie folgend (starker Konsens), die Entbindung in einer dafür geeigneten Einrichtung unter Bereitstellung der notwendigen Ressourcen geplant werden. Hierzu zählen neben der personellen Expertise auch logistische Voraussetzungen wie adäquate venöse Zugänge, die Bereitstellung von Uterotonika (Oxytocin/Carbetocin, Sulproston), Tranexamsäure und Gerinnungsprodukten. Auch ein Notfalllabor (Blutbild, Blutgasanalyse [BGA], aPTT, Quick-Wert bzw. INR und – sofern verfügbar – Fibrinogen, Faktor XIII, viskoelastische Tests [VET]) und eine Blutbank/ein Blutdepot sollen vorhanden sein.

Es wird empfohlen, alle Aspekte in einem auf die jeweiligen Bedingungen der einzelnen Klinik abgestimmten Behandlungsalgorithmus für die peri-/postpartale Blutung, den alle Krankenhäuser mit geburtshilflicher Abteilung erstellen sollen, interdisziplinär zusammenzufassen.

Gerade PAS gehen sehr häufig mit schwerster PPH einher, sodass hier die Entbindung „zum optimalen Zeitpunkt vom optimalen Team“ durchgeführt werden sollte. Auch die Schwangere im Fallbeispiel weist mit einem mütterlichen Alter über 30 Jahre, einem BMI > 35 und Multiparität bereits 3 Risikofaktoren für die Entwicklung einer PPH auf. Sowohl nach vaginaler Entbindung im Rahmen einer „aktiven Leitung der Nachgeburt“ als auch nach Kaiserschnitt kommt der medikamentösen Prophylaxe einer PPH eine besondere Bedeutung zu. Hierzu wird die i.v.-Kurzinfusion von Oxytocin 3–5 IE oder Carbetocin 100 µg empfohlen. Bolusgaben sind aufgrund vermehrter kardiovaskulärer Nebenwirkungen zu unterlassen. Carbetocin zeigt bei gleicher Nebenwirkungsrate aufgrund einer längeren Halbwertszeit eine länger anhaltende Wirksamkeit. Methylergometrin ist aufgrund vermehrter Nebenwirkungen ebenso wie Misoprostol (dieses aufgrund im Vergleich zu Oxytocin verminderter Wirksamkeit) kein Mittel der ersten Wahl zur Prophylaxe einer PPH. Tranexamsäure soll aufgrund einer unzureichenden Wirksamkeit nicht prophylaktisch eingesetzt werden.

## Kasuistik: postpartale Hämorrhagie

**Figure 1:**
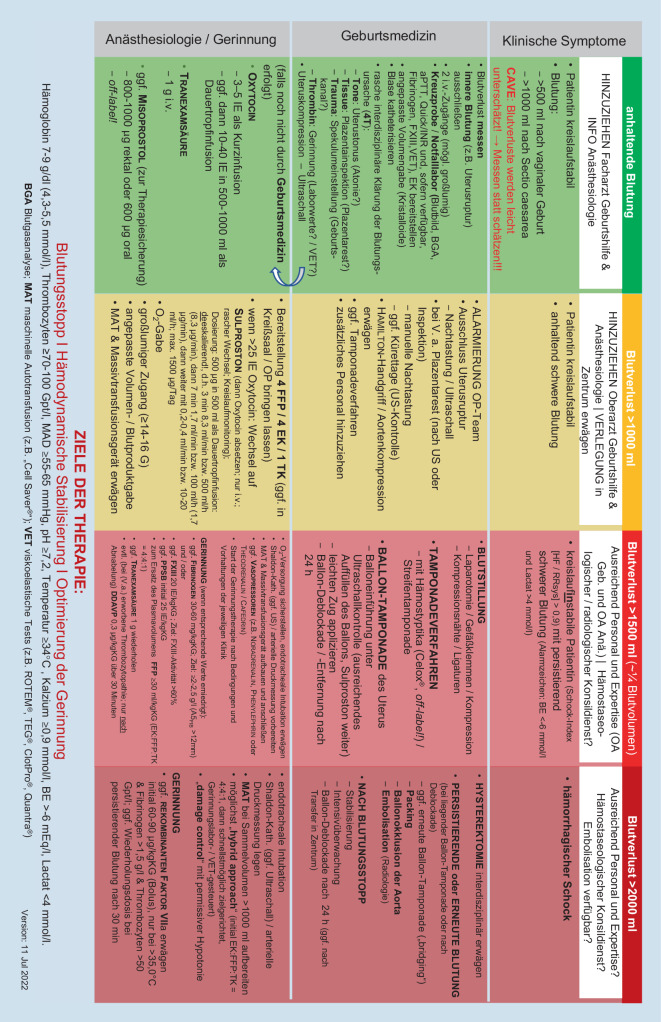

*Die Blutung nach Abschluss der Geburt erscheint verstärkt. In den angebrachten Sammelbeuteln konnten 1000* *ml aufgefangen werden. Zusätzlich gibt es zahlreiche blutgetränkte Kompressen, sodass durch die Geburtshelfer ein Gesamtblutverlust von 1200–1300* *ml geschätzt wird. Die Vitalparameter der Mutter sind stabil. Bei Diagnose PPH werden das anästhesiologische Dienstteam und die geburtshilfliche Oberärztin informiert und weitere 40* *IE Oxytocin in 1000* *ml Vollelektrolytlösung als Dauertropfinfusion sowie 1* *g Tranexamsäure verabreicht und der Uterus manuell komprimiert. Eine laminierte Version des PPH-Algorithmus (*Abb. 1*) wird im Kreißsaal bereitgelegt und im Rahmen eines „Team-Time-Out“ das Vorliegen einer „PPH mit anhaltend schwerem Blutverlust, jetzt bereits mehr als 1000* *ml“, und das geplante weitere Vorgehen nach Algorithmus besprochen. Durch das anästhesiologische Team wird ein zweiter venöser Zugang gelegt, Blut für die Standardlaboranalyse und die viskoelastische Testung (VET) abgenommen sowie die Blutbank telefonisch über die Situation vorab informiert. Zeitgleich evaluieren Hebamme und geburtshilfliches Team systematisch die Ursache(n) für die persistierende Blutung unter Berücksichtigung sämtlicher Hauptursachen „tonus, tissue, trauma, thrombin“*.

## Leitlinie: Definition und Pathophysiologie einer PPH und Erstmaßnahmen mit Ursachensuche

**Figure 2:**
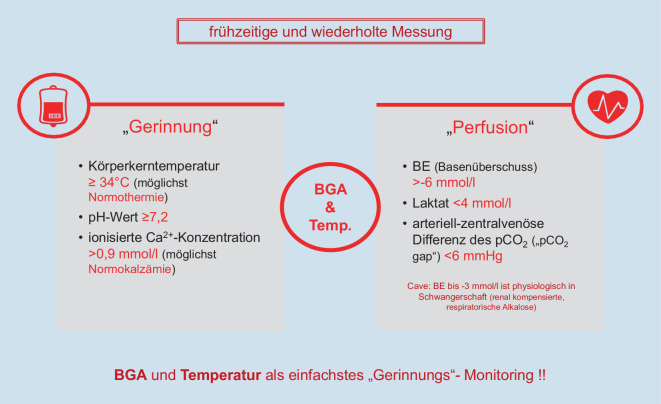

Die physiologischen schwangerschaftsbedingten Anpassungsvorgänge bereiten die werdende Mutter gut auf moderate Blutverluste vor. Blutverluste werden visuell meist unterschätzt, sodass in der aktualisierten Leitlinie validierte Messverfahren empfohlen werden. Per Definition liegt eine verstärkte Blutung vor, sofern nach vaginaler Entbindung ≥ 500 ml und nach Sectio caesarea mehr als ≥ 1000 ml Blut verloren werden. Neben der Messung des Blutverlustes soll zur Entscheidungsfindung engmaschig der klinische Zustand der Patientin beurteilt werden. Ein positiver Schockindex (Herzfrequenz/systol. Blutdruck) > 0,9, eine metabolische Acidose und erhöhte Lactatwerte können auf eine kritische Hypovolämie hinweisen. Hingegen reagiert der Hb-Wert häufig erst verzögert. Eine PPH führt zu einem hämorrhagischen und nicht zu einem traumatisch-hämorrhagischen Schock, da der Gewebeschaden, verglichen z. B. mit einem Polytrauma, deutlich geringer ist. Die Blutgerinnung ist daher initial meist noch intakt, sofern keine angeborene Gerinnungsstörung, Medikamentenwirkung oder eine Fruchtwasserembolie vorliegt. Kann die Blutung nicht schnell kontrolliert werden, kann sich im Verlauf durch Verlust, Verbrauch, schockbedingte Endotheliopathie und iatrogene Verdünnung sowie eine einsetzende Hypothermie eine koagulopathische Blutung entwickeln. Nach aktueller Evidenz soll bei Stellung der (Verdachts‑)Diagnose PPH frühzeitig und parallel zum therapeutischen Oxytocin Tranexamsäure gegeben werden, da dies zumindest Blutverlust und Transfusionsbedarf positiv beeinflussen kann [3]. Daneben müssen frühzeitig die Rahmenbedingungen für eine adäquate Hämostase (pH, Temperatur, Kalzium) aufrechterhalten bzw. wiederhergestellt werden, und die Infusionstherapie zur Schockbekämpfung soll streng bedarfsadaptiert unter Vermeidung einer Überinfusion erfolgen (Abb. 2). Oberste Priorität haben die Identifikation der Blutungsquelle und die Blutstillung. Durch Anamnese, klinische Untersuchung, Nachtastung und insbesondere repetitive Ultraschalluntersuchungen müssen die häufigen Blutungsursachen, die 4 T, Atonie (Tonus), Geweberetention (Tissue), Geburtsverletzungen, parametrane Blutungen und Uterusruptur (Trauma) sowie angeborene oder erworbene Gerinnungsstörungen (Thrombin), ausgeschlossen werden. Parallel soll durch Uterotonika und physikalische Maßnahmen wie manuelle Uteruskompression der Versuch einer Blutstillung erzielt werden.

## Kasuistik: schwere Atonie

*Der Uterus erscheint trotz Oxytocininfusion weiterhin atonisch. Klinische und sonographische Untersuchung sowie eine Nachtastung ergeben keine Hinweise auf eine Plazentaretention, Verletzung des Geburtskanals oder eine erworbene oder vorbestehende Gerinnungsstörung. Es wird daher die Oxytocininfusion gestoppt und eine Dauertropfinfusion mit Sulproston gestartet. Bei persistierender Blutung entscheidet das Team zur weiteren Versorgung der Patientin in den unmittelbar angrenzenden Sectio-OP umzuziehen. Eine interventionelle Radiologie ist im Haus nicht verfügbar. Es werden 4 Erythrozytenkonzentrate sowie 4 FFP in den Sectio-OP bestellt und ein Thrombozytenkonzentrat auf Abruf geordert*.

## Leitlinie: Blutstillungsmaßnahmen

Uterotonika sind die Grundlage der medikamentösen Blutstillung bei PPH (Abb. 1), da sie die endogene Kompression des Uterus fördern. Bei persistierender Blutung müssen umgehend die personellen und logistischen Voraussetzungen zur Versorgung der Patientin optimiert werden. Hierzu zählen auch Vorbereitungen für weitere konservative oder operative Blutstillungsmaßnahmen wie Tamponadeverfahren, Laparotomie, Kompressionsnähte, ggf. Hysterektomie. Sofern verfügbar sollen auch (radiologisch-) interventionelle Verfahren zur Blutungskontrolle erwogen werden (Embolisationen, Ballonokklusionen, temporäre Aortenokklusion). Neben der nichtvorhandenen nötigen Expertise limitiert jedoch auch die häufig nicht mehr gegebene Kreislaufstabilität der Patientin den Transport in eine radiologische Abteilung und den Einsatz dieser Verfahren in vielen Krankenhäusern. Neben dem Erfolg der Maßnahmen zu Schockbekämpfung, Gerinnungsoptimierung und Blutungskontrolle müssen, ähnlich der Polytraumaversorgung, alle Ursachen einer persistierenden Blutung regelmäßig reevaluiert werden.

## Kasuistik: hämorrhagischer Schock

*Die Patientin erscheint zunehmend tachykard und kaltschweißig. Der geschätzte Blutverlust beträgt >* *2500* *ml. Sie hat mittlerweile 2500* *ml Vollelektrolytlösung erhalten. Eine aktuell gewonnene venöse Blutgasanalyse zeigt nur noch einen Hb-Wert von 7,3* *g/dl, einen Base Excess von −6,5* *mmol/l und ein Lactat von 5* *mmol/l. Bei hämorrhagischem Schock und weiter persistierender Blutung wird im Team entschieden, bei der Patientin eine Allgemeinanästhesie einzuleiten und eine endotracheale Intubation durchzuführen. Die Patientin erhält eine arterielle Blutdruckmessung und einen Zentralvenenkatheter. Es wird eine maschinelle Autotransfusion (MAT) aufgebaut und ein kontinuierlicher Wärmeerhalt sichergestellt. Darüber hinaus erfolgt eine Information an die Blutbank über die persistierende Blutung. Eine erneute Evaluation möglicher Blutungsquellen (4* *T) ergibt außer einer weiter bestehenden Atonie keine neuen Erkenntnisse. Bei persistierender Blutung wird im Team ein Vorgehen nach „Damage-control“-Prinzipien beschlossen und der Versuch einer temporären Blutstillung durch die Insertion eines Bakri-Ballons zu erreichen unternommen. Parallel erhält die Patientin eine Antibiotikaprophylaxe und bedarfsadaptiert Erythrozytenkonzentrate, FFP und TK nach einem Massivtransfusionsprotokoll im Verhältnis 4:4:1. Eine operativ besonders erfahrene geburtshilfliche Oberärztin und der leitende Oberarzt der Anästhesieabteilung werden über die Situation einer evtl. drohenden Hysterektomie informiert und begeben sich in den Kreißsaal*.

## Leitlinie: Schockbekämpfung

Ähnlich wie in der Traumaversorgung hat die Blutungskontrolle zur Schockbekämpfung auch bei einer PPH oberste Priorität. Um eine Gewebehypoxie zu vermeiden, muss ein situativ angemessener Volumenersatz unter Vermeidung einer iatrogenen Verdünnung durchgeführt werden. Hierzu müssen geeignete Zugänge gelegt und initial ein Volumenersatz mit kristalloiden Lösungen begonnen werden. Temporär kann ein „Damage-control-resuscitation“-Ansatz mit Inkaufnahme niedriger Blutdruckwerte (systolischer Blutdruck ~ 80 mm Hg) sinnvoll sein, um eine schnellere Blutstillung zu erzielen. Im Einklang mit der S3-Leitlinie zur Volumentherapie des Erwachsenen [4] sollen klinische Zeichen und weitere Hinweise auf einen fortbestehenden Volumenmangel („passive leg raising“, Reaktion auf Volumenbolus, Volumenreagibilität bei beatmeten Patienten, eFAST) zur Steuerung der Volumentherapie genutzt werden. Bei schwerer und anhaltender Blutung ist ein an der Vollblutzusammensetzung orientiertes Massivtransfusionsregime (EK:FFP:TK = 4:4:1) sinnvoll. Auch sollte die Nutzung der MAT in Abhängigkeit von den lokalen Gegebenheiten erwogen werden.

## Kasuistik: Damage control

*Die Patientin wurde bei persistierender schwerer Blutung mittlerweile laparotomiert. Um die Blutung unter Kontrolle zu bringen, wurden Uteruskompressionsnähte angelegt sowie Gefäßklemmen gesetzt. Eine erneut abgenommene Point-of-care-Gerinnungsanalyse (POCT-Analyse) zeigt jetzt eine verlängerte Blutungszeit sowie eine verminderte Gerinnselfestigkeit mit Fibrinogenmangel. Die erweiterte Gerinnungsanalyse ergibt darüber hinaus eine deutlich verminderte Faktor-XIII-Aktivität. Beide Gerinnungsfaktoren werden daraufhin substituiert, und es werden noch einmal alle Grundvoraussetzungen für die Hämostase optimiert. Trotz aller konservativen Maßnahmen kann die Uterusatonie weiter nicht suffizient kontrolliert werden. Die temporäre Blutstillung ermöglichte jedoch eine suffiziente Schocktherapie und Gerinnungsstabilisierung, sodass nach Eintreffen der geburtshilflichen Oberärztin und des leitenden Oberarztes Anästhesie (beste mögliche Expertise) nach Risikoabwägung eine Hysterektomie durchgeführt wird. Zur Hysterektomie sind weitere Transfusionen und eine labor- bzw. POCT-gesteuerte gezielte Substitution von weiteren Gerinnungsprodukten erforderlich*.

## Leitlinie: „Damage control“ und Gerinnungstherapie

Führen konservative Blutstillungsmaßnahmen nicht zum Erfolg, müssen rechtzeitig operative Schritte folgen. Auch eine notwendige Hysterektomie sollte nicht zu spät indiziert werden. Da dieser Eingriff zeitaufwendig ist und mit weiterem Blutverlust einhergeht, ist es ratsam, zunächst eine temporäre Blutstillung zu erreichen und die Patientin vor der Hysterektomie zunächst hämodynamisch zu stabilisieren und die Gerinnung zu optimieren.

Eine im Rahmen der Entbindung auftretende Koagulopathie bei PPH ist als erworben anzusehen. Die hämostaseologische Standardtherapie ist die frühzeitige Gabe von Tranexamsäure, sobald die Diagnose der PPH gestellt worden ist. Daneben ist der Aufrechterhaltung optimaler Rahmenbedingungen (Temperatur, Kalzium, pH-Wert, Hb-Wert) besondere Aufmerksamkeit zu schenken. Indikationen für Gerinnungsfaktoren bzw. Thrombozytenkonzentrate sind nach der neuen Leitlinie das Vorliegen einer anhaltend schweren peripartalen Blutung und der Nachweis eines Mangels an Gerinnungsfaktoren oder Thrombozyten. Formelbasierte oder verhältnisorientierte Substitution erscheint nicht sinnvoll, da längst nicht alle Patienten sofort einen Mangel an Gerinnungsfaktoren haben und die präventive Gabe von Fibrinogen keine Reduktion von Blutverlust oder Transfusionsbedarf erzielt hat. Zu diesem Zweck ermöglichen eine VET und die Diagnostik im Standardlabor die gezielte Identifikation und dann auch Substitution von bestehenden Gerinnungsfaktorenmängeln. Dies ist von Bedeutung, da unterschiedliche Ätiologien der PPH (z. B. PAS vs. vorzeitige Plazentalösung vs. Trauma vs. …) zu unterschiedlichen VET-Ergebnissen führen und somit die VET zeigt, was substituiert werden und wann nicht substituiert werden muss.

Bei schwerer PPH mit anhaltenden Blutungen ist im Einklang mit den europäischen Leitlinien zunächst ein „hybrid approach“, d. h. initial eine verhältnisgesteuerte Therapie mit EK und FFP und/oder Gerinnungsfaktoren, dann schnellstmöglich gefolgt von einer zielgerichteten POC-gesteuerten Therapie mit Blutprodukten und Gerinnungsfaktoren, zu empfehlen. Hierbei ist auf einen ausgewogenen Einsatz aller beteiligten Komponenten bei nachgewiesenem oder antizipierendem Mangel zu achten. Begründbare Ziele finden sich für Thrombozyten 70–100 G/l, Fibrinogen > 2–2,5 g/l und FXIII > 60 % [5–10]. Bevor prokoagulante Faktoren gegeben werden, muss immer eine möglicherweise bestehende erhöhte fibrinolytische Aktivität durch Tranexamsäure behandelt werden. Es gibt keine Datenbasis zum Einsatz von DDAVP (Desmopressin) bei der PPH. Indikationen sind somit lediglich die Thrombopathien, bei denen eine DDAVP-Response gezeigt werden konnte oder diese anzunehmen ist. DDAVP sollte, wenn indiziert, zur Vermeidung von Nebenwirkungen beim Fetus immer erst nach der Abnabelung verabreicht werden. Wie schon in der vorangegangenen Version der Leitlinie gilt rekombinanter FVIIa als „Rescue“-Therapie nur nach Einzelfallentscheidung und individueller Nutzen-Risiko-Abwägung sowie vorheriger Ausschöpfung anderer Methoden zu Blutstillung, Gerinnungsoptimierung und Wiederherstellung der Rahmenbedingungen für eine adäquate Hämostase. Nach Abschluss der Konsensusfindung zur Leitlinie wurde durch die Europäische Arzneimittelbehörde die Indikation für rFVIIa auf die „*schwere postpartale Hämorrhagie, wenn Uterotonika* (inklusive Sulproston)* nicht ausreichen, um eine Hämostase zu erreichen“ erweitert. *Die Publikation der zugrunde liegenden Daten steht noch aus, sodass durch die Leitliniengruppe keine Neubewertung von Nutzen und Risiken durchgeführt werden konnte. Rekombinanter Faktor VIIa ist somit auch nach dieser Neuerung keinesfalls als frühzeitige Routinetherapie anzusehen.

## Kasuistik: weitere Therapie

*Am Ende der Operation ist die Patientin kreislaufstabil, aber trotz aller Wärmemaßnahmen noch hypotherm, sodass sie beatmet auf die anästhesiologische Intensivstation verlegt wird. Kurze Zeit später kann sie extubiert werden. Bei fehlenden Blutungszeichen und stabiler Gerinnung wird bereits am Abend mit einer prophylaktischen Antikoagulation begonnen. Die Patientin und ihre Angehörigen werden ausführlich über das Krankheitsbild PPH informiert. Auch das Team führt ein interdisziplinäres Debriefing durch und nimmt den Fall zum Anlass, einen Termin für ein nächstes interdisziplinäres Simulationstraining festzulegen*.

## Leitlinie: Anschlusstherapie

Nach einer PPH ist die Patientin adäquat zu überwachen. Innerhalb von 24 h nach Beendigung der Blutung soll eine Thromboseprophylaxe durchgeführt werden. Bei Vorliegen von Risikofaktoren ist diese bis zu 6 Wochen postpartal fortzusetzten. Bei verminderter Antithrombinaktivität kann eine Substitution auf der Intensivstation nach Ende der Blutung erwogen werden. Auch sollte bei Nachweis eines blutungsbedingten Eisenmangels eine Eisensubstitution durchgeführt werden. Interdisziplinäre und interprofessionelle Nachbesprechungen und Teamtrainings dienen der adäquaten Vorbereitung auf das häufige Notfallereignis PPH.

## Fazit für die Praxis

- Peripartale Blutungen sind in der Praxis häufig und treten oft ohne vorbestehende Risikofaktoren auf.
- Vorhandene Risikokonstellationen müssen erkannt werden.
- Eine medikamentöse aktive Leitung der Nachgeburtsperiode ist zur Prophylaxe einer PPH wichtig.
- Blutverluste werden meist unterschätzt.
- Bei Diagnosestellung einer PPH soll frühzeitig Tranexamsäure verabreicht werden.
- Zur Therapie der PPH muss sofort eine Ursachenabklärung (4 T) durchgeführt werden, um eine gezielte Blutungskontrolle zu erreichen.
- Volumen soll zur Schocktherapie bedarfsadaptiert substituiert werden.
- Ggf. ist ein Massivtransfusionsprotokoll zu nutzen (EK:FFP:TK = 4:4:1).
- Die Indikation für die Gabe von Gerinnungsprodukten ist die persistierende Blutung und der nachgewiesene Mangel.
- Bei der Versorgung der Patientin sind ggf. „Damage control“-Konzepte zu nutzen.
- Zur Behandlung ist die „beste mögliche verfügbare Kompetenz“ sicherzustellen.
- Ein Debriefing für das Team und auch die Patientin bzw. das Paar soll sichergestellt werden.
- Bereits im Vorfeld sind interdisziplinäre und interprofessionelle Trainings zu etablieren.
